# Anthropogenic Object Localization: Evaluation of Broad-Area High-Resolution Imagery Scans Using Deep Learning in Overhead Imagery

**DOI:** 10.3390/s23187766

**Published:** 2023-09-08

**Authors:** J. Alex Hurt, Ilinca Popescu, Curt H. Davis, Grant J. Scott

**Affiliations:** 1 Center for Geospatial Intelligence, University of Missouri, Columbia, MO 65211, USA; 2 Department of Geography, Stanford University, Stanford, CA 94305, USA; 3 Department of Electrical Engineering and Computer Science, University of Missouri, Columbia, MO 65211, USA

**Keywords:** broad-area scan, deep convolutional neural network (DCNN), object detection

## Abstract

Too often, the testing and evaluation of object detection, as well as the classification techniques for high-resolution remote sensing imagery, are confined to clean, discretely partitioned datasets, i.e., the closed-world model. In recent years, the performance on a number of benchmark datasets has exceeded 99% when evaluated using cross-validation techniques. However, real-world remote sensing data are truly big data, which often exceed billions of pixels. Therefore, one of the greatest challenges regarding the evaluation of machine learning models taken out of the clean laboratory setting and into the real world is the difficulty of measuring performance. It is necessary to evaluate these models on a grander scale, namely, tens of thousands of square kilometers, where it is intractable to the ground truth and the ever-changing anthropogenic surface of Earth. The ultimate goal of computer vision model development for automated analysis and broad area search and discovery is to augment and assist humans, specifically human–machine teaming for real-world tasks. In this research, various models have been trained using object classes from benchmark datasets such as UC Merced, PatternNet, RESISC-45, and MDSv2. We detail techniques to scan broad swaths of the Earth with deep convolutional neural networks. We present algorithms for localizing object detection results, as well as a methodology for the evaluation of the results of broad-area scans. Our research explores the challenges of transitioning these models out of the training–validation laboratory setting and into the real-world application domain. We show a scalable approach to leverage state-of-the-art deep convolutional neural networks for the search, detection, and annotation of objects within large swaths of imagery, with the ultimate goal of providing a methodology for evaluating object detection machine learning models in real-world scenarios.

## 1. Introduction

The detection of objects in high-resolution (HR) electro-optical (EO) remote sensing imagery (RSI) has been studied for many years. Objects in RSI are commonly referred to as anthropogenic features on the surface of the Earth, which are either movable or structural entities. Numerous applications will benefit from improved techniques to recognize and localize objects in HR-EO-RSI, such as urban monitoring and management, mapping applications, area searches, and many more. A critical need exists to increase the level of visual search automation to allow humans to effectively manage the deluge of overhead sensor data that are being continuously collected by overhead imagery sources, such as satellites and drones.

At this point, there exist numerous datasets of HR-EO-RSI, including the UC Merced dataset [1], the RSD or WHU-RS19 [2], the PatternNet [3], the RSI-CB256 [4], the AID [5], and the RESISC-45 [6]. These datasets have supported a wide variety of land cover classification and object detection research since their releases over the last ten-plus years. The research has explored many approaches, which range from hand-crafted features fed into many types of classifiers to comprehensive deep learning models.

Many of these datasets have been released with initial studies of the classification performance, such as [4,5,6], that routinely compare the performance of hand-crafted features to deep learning techniques. In each case, deep learning methods have demonstrated superior classification performance and object detection accuracies. Specifically, deep convolutional neural networks (DCNNs) have emerged as leading approaches that have demonstrated excellent performance in cross-validation studies [7,8] and other studies utilizing discrete remote sensing datasets [9,10,11,12]. This has continued as vision transformers [13] and other transformer-based models have been utilized to further improve the classification performance of HR-EO-RSI [14,15]. While some studies do choose to utilize DCNNs on real-world remote sensing imagery, they often choose to investigate small areas where the ground truth is readily available [16,17,18]. However, rarely does published research move beyond the cross-validation of these datasets and small investigation areas to evaluate object localization with DCNNs that deal with broad areas of aerial imagery. That is, models are only evaluated in closed-world settings, and not enough research has been performed on DCNN models trained on discrete datasets in the larger context of Earth observation.

It is not well understood how well these models perform in the complex realities of broad-area searches (BASs) of HR-EO-RSI. Real-world remote sensing data are truly big data, and they often exceed billions of pixels in a single scene capture. To apply DCNN models, practitioners need algorithmic approaches for scanning data at an appropriate range of ground sample distance (GSD) (e.g., 0.5 m GSD, 1.0 m GSD, etc.). Detection results must then be post-processed to be filtered and refined, thereby generating an ordered list of likely object locations for human consumption. The sheer volume of HR-EO-RSI that is collected prevents an exhaustive human effort to label data, which both necessitates the use of machine learning models and precludes solely relying on ground truth labels as the only evaluative method for those models.

Scanning, i.e., sliding sub-image analysis, large swathes of Earth imagery with DCNN models produces a classification vector response field (CVRF), which is a multi-class response of the center point of the DCNN input field of view. The CVRF can be handled in a number of ways, with the most straightforward being to extract layers (i.e., a single class of object detections). By their very nature, DCNNs are somewhat robust in response to the shift and rotation of objects in HR-EO-RSI. Therefore, if the scanning operation has a suitable overlap of image chips that are fed to the DCNN, we can expect a single instance of an object to be detected to varying degrees in multiple nearby locations. The challenge is, then, to reduce the CVRF into a set of salient object locations and, furthermore, to rank order these locations for human analytical consumption. However, evaluating machine learning models taken out of the clean laboratory setting and into the real world is difficult due to the lack of massively scaled ground truth data at the scale of an operational remote sensing task.

The novel contributions of this paper include investigation into the translation of DCNN cross-validation performance into BAS applications. Additionally, we present a metric for assessing the performance of a DCNN detector in BAS applications that accurately scores models in situations with and without *a priori* ground truth labels. This metric, known as scanning precision (SP), returns a real number between 0 and 1, thus enabling direct comparisons between detectors and functions by emphasizing true detections where the model is most confident. It strongly penalizes models for having high confidence in false positives, and it penalizes models less for false positives that have lower confidence. Finally, this paper performs BASs with multiple trained DCNN models on multiple AOIs and processes a total area of 50,000 square kilometers. We then evaluate the BAS performance using the proposed SP metric to investigate the ability of deep learning models to perform machine-assisted visual analytics, and we thereby enable the faster and more efficient detection of objects by assisting humans in finding the desired objects in BAS tasks.

The remainder of this paper is organized as follows. Section 2 discusses the training methodologies used to build BAS capability, including the datasets and network architectures used for generating trained models, as well as the algorithmic approach for object detection localization in the context of broad-area scans. Section 3 then contains our experimental results and discussion, starting from base network performance with respect to the examined datasets in cross-validation and then presenting the results and discussion of the broad-area search performance using the proposed scanning precision (SP) metric to quantitatively evaluate and compare the scanning results. Finally, we conclude with some final remarks and future directions for this research in Section 4.

## 2. Materials and Methods

To generate the models used for BASs, we considered multiple architectures and datasets. Modern DCNNs vary greatly in their architectures, and these differences may be crucial in object detection performance in highly variable regions of Earth. Additionally, the choice of augmentation and other hyperparameters such as learning rate or momentum can affect how the DCNN performs in a BAS task. We considered four benchmark datasets for this research, which are detailed in the next subsection. Furthermore, we evaluated three network architectures, which are detailed in Section 2.4.2: EfficientNet, ProxylessNAS, and Xception. These three networks utilize diverse feature extraction techniques, and by evaluating all three against the selected datasets, we will be able to better understand the performance of the models in cross-validation versus scanning performance when considering the differing characteristics of the models.

### 2.1. Datasets

**Figure 1:**
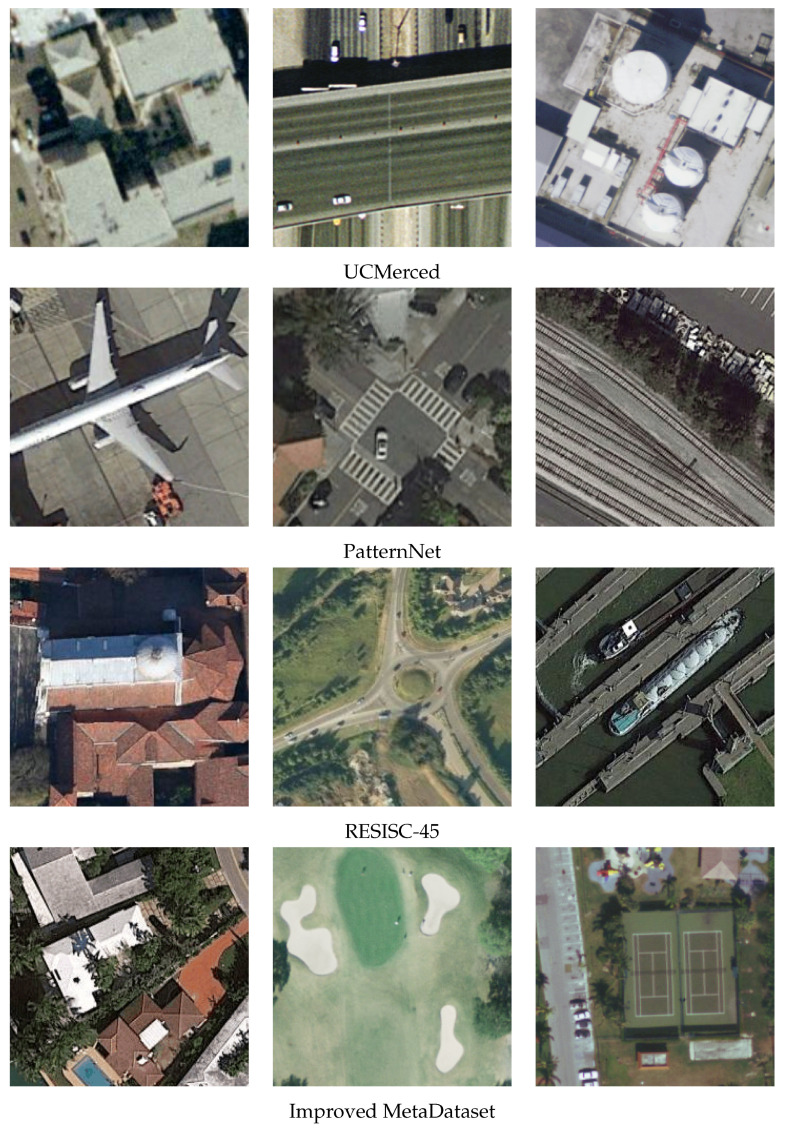
Sample imagery from the four chosen datasets: UCMerced, PatternNet, RESISC-45, and Improved MetaDataset.

In recent years, many benchmark datasets have been released, and these datasets have been researched with a high level of success, such as in [8,19,20,21]. With these datasets, a high level of average recall can be obtained through the careful selection of hyperparameters and augmentation schemes. For this research, we will evaluate the performance of DCNN models regarding their ability to detect and locate five selected anthropogenic features in large swaths of aerial imagery: *Airplane*, *Baseball Diamond*, *Overpass*, *Storage Tanks*, and *Tennis Courts*. We chose four benchmark datasets containing these five classes for the training of our DCNN models for eventual use in BAS. These datasets are described below, and sample image chips from each dataset can be seen in Figure 1.

#### 2.1.1. UC Merced

The UC Merced (UCM) benchmark dataset [1] has been used in a wide range of remote sensing research, which includes prior work in the classification of objects and land cover, such as [7,8,22,23,24,25]. It includes 21 classes with 100 samples each. This dataset is well standardized and has consistent resolution of 256 × 256 pixel images of 0.3 m GSD. As shown in Table 1, column **UCM**, UC Merced contains all five classes of interest for BAS, with each containing the standard 100 images.

#### 2.1.2. PatternNet

The PatternNet benchmark dataset was designed by Zhou et al. [3] for remote sensing image retrieval. This dataset includes 38 classes, each with 800 image samples. The image chips are 256 × 256 pixels, and the spatial resolution varies from 0.06 m to 4.7 m GSD. All five of the classes of interest for BAS are included in the PatternNet dataset, as shown in Table 1, column **PN**.

#### 2.1.3. NWPU-RESISC45

The Remote Sensing Image Scene Classification 45 (RESISC-45) is a benchmark remote sensing dataset developed by Cheng et al. [6]. It was designed to be a challenging remote sensing image scene classification benchmark dataset. It contains 45 classes, each with 700 samples. The image chips are 256 × 256 pixels, and the spatial resolution varies from 0.2 m to 30 m GSD. RESISC-45 is the most difficult dataset to classify in cross-validation evaluation, as shown in [19]. This dataset’s selected class distribution is shown in Table 1, column **R-45**.

#### 2.1.4. Improved Benchmark Meta-Dataset

The Improved Benchmark Meta-Dataset (MDSv2) [26] was developed in 2020 as an enhanced version of the original Benchmark Meta Dataset (MDS) [27]. The MDSv2 combines co-occurring and unique classes from six distinct overhead datasets, including four benchmark datasets and two challenge datasets. The resulting dataset is heavily unbalanced, with as little as 700 samples and as many as 17,000 in the largest class. It has over 80,000 samples belonging to 33 classes and is the dataset with the most intra-class diversity in this study where GSD and visual features are concerned. The dataset was designed with BAS in mind, and as such, one would expect it to train the most robust detectors in our BAS experiments. This dataset’s class distribution is shown in Table 1, column **MDSv2**.

#### 2.1.5. Ground Truth Datasets

Ground truth was collected in the two areas of interest (AOIs) of Beijing, CHN, and Springfield, MO, USA. All ground truth was collected by utilizing Google Earth Pro. The larger Beijing AOI was divided into 9 smaller sections for purposes of ground truth collection. For each section, the area was scanned horizontally for target objects of (1) airplanes, (2) storage tanks, and (3) street overpasses, for a total of 3 layers for each subsection. When an object of interest was found, a pin was placed, and the object was labeled. Each area was then given a second pass to ensure all objects were correctly labeled and that no object of interest had gone unlabeled.

Ground truth collection was timed to enable comparison between human-alone and machine-assisted methodologies for detecting these objects of interest. This same methodology was utilized for Springfield, MO, but it utilized 12 smaller polygons to divide the AOI rather than the 9 used in the Beijing AOI. The data was then exported to KML and later used for computing performance metrics of models in detecting objects of interest in the 2 AOIs. The total number of objects collected from the manual review of our AOIs is 1826 across the three chosen classes, with a total of 1471 objects in the Beijing AOI and 355 in Springfield. More details on the number of objects collected per class in each AOI are available in Table 2.

The total number of hours required to collect the ground truth data was 113:44:32, with around 72 h allocated to the smaller Springfield AOI and around 42 h for data collection in the Beijing AOI. In both AOIs, the class requiring the most time to collect ground truth was the Overpass class; however, the Springfield AOI required more than 2× the human hours to collect Overpass ground truth, despite having only 1/4th the area of review. This trend continued for the Airplane class, as the smaller Springfield AOI required more collection time at 32 h than the larger Beijing AOI at 12.5 h. We then observed the opposing trend in the data collection times for the Storage Tank class, as the Springfield AOI only required 3.5 h for data collection, while the larger Beijing AOI required just under 4× the time at 13 h. As noted, the manual collection of data from the imagery required over 100 h of human eyes on imagery. This is ultimately the task we seek to enable with human–machine teaming, thereby leveraging the trained networks for BAS to reduce the human time element by one to two orders of magnitude.

### 2.2. Network Architectures

In the sections below, we describe the deep network architectures that we utilized in this study. We chose three architectures with differing design paradigms so that we could evaluate multiple types of deep networks in BAS applications.

#### 2.2.1. EfficientNet

The EfficientNet [28] family of networks was developed in 2019 and was presented at the International Conference of Machine Learning (ICML). EfficientNet is a family of 8 different architectures, where 7 of them are scaled networks from the original network: EfficientNet-B0. EfficientNet-B0 is the result of neural architecture searches (NASs), but, unlike other NAS-based networks, EfficientNet-B0 optimizes both to maximize network accuracy, while also minimizing FLOPS, i.e., it produces a highly efficient and well-performing network. After establishing the architecture of the EfficientNet-B0, the authors scaled the network using a compound scaling coefficient, ϕ, to scale the depth, width, and resolution of the network to larger sizes. The resulting family of networks offers eight options, each with a different level of tradeoff between the number of parameters and the ImageNet accuracy. For this research, we used EfficientNet-B4, which is a model with ImageNet performance similar to NASNet-A [29] but with only 4.2B FLOPs, which is similar to ResNet-50 [30] in the number of FLOPs and which has 5.7 × fewer FLOPs than similarly performing networks, e.g., NASNet-A.

#### 2.2.2. ProxylessNAS

The ProxylessNAS network [31] advertises better performance, lower training times, and smaller memory footprints than NASNet-A [29]. When compared on the CIFAR-10 dataset [32], the ProxylessNAS architecture was able to achieve a 2.08% test error, which was better than NASNet-A’s respectable 2.40% test error. One important note on these results, however, is that the NASNet-A has 27.6 million learnable parameters, while ProxylessNAS has only 5.7 million. With regard to training performance, ProxylessNAS not only outperforms NASNet-A’s 38.3 ms GPU latency, but its 5.1 ms GPU latency is faster than ResNet-34 (8.0 ms) or MobileNetV2 (6.1 ms) when used on the NVIDIA Tesla V-100. The times reported are taken from an experiment on the ImageNet dataset [33] published in the International Conference on Learning Representations (ICLR) paper for ProxylessNAS. What is more, these times do not come at the cost of classification performance, as the ProxylessNAS has a 92.5% Top-5 accuracy on ImageNet, which again has outperformed NASNet-A (91.3%) and ResNet-34 (91.4%). While ProxylessNAS has shown promise, there are still questions as to how ProxylessNAS will perform on HR-EO-RSI.

#### 2.2.3. Xception

Xception is another popular network that has achieved a high level of success in the remote sensing domain [19]. The Xception architecture is one that relies on residual connections similar to ResNet-50 but adds an extreme inception module to the residual layers. The extreme inception module is based on the inception module used in the Inception networks, specifically, the inception module used in Inception V-3’s architecture [34]. This module creates a pseudo-depth-wise separable convolution that performs better than standard convolutional layers in many contexts. Important characteristics of this network are 36 residual, depth-wise separable convolutional layers with ReLU activation functions that are separated into 14 modules before being fed into a single fully connected layer with a softmax output.

### 2.3. DCNN Training Methodology

We now detail our methodology for training DCNN models for both our cross-validation experiments as well as for the BASs. The training methodology follows the prescribed concepts of transfer learning and data augmentation described in [7]. Regarding the hyperparameters, all models were trained using an Adam optimizer and a categorical cross-entropy loss function with an initial learning rate of 0.0001 ($1\times 10^{-4}$). All networks were initialized with publicly available ImageNet [33] weights (transfer learning) and are GPU-accelerated with four NVIDIA V100S running the PyTorch deep learning framework in Python 3.6.

#### 2.3.1. Training DCNNs for Cross-Validation

As shown in [7], data augmentation can significantly improve the performance of DCNNs. For the four datasets considered here, augmentations applied to the training image chips included rotations and horizontal flips. Conceptually, these augmentations are increasing the variability of the data the models are exposed to during training, with the goal of increasing generalizability. The augmentation through rotation was selected to be in increments of five degrees from 0 to 360. All four datasets received identical augmentation schemes, and the total data augmentation multiplier during cross-validation was 144. The training length was set to only a single epoch; however, it should be noted that, while we only trained for a single epoch, our use of data augmentation means we effectively were training for 144 epochs with randomly shuffled variations in our original dataset.

#### 2.3.2. Training DCNNs for Scanning

The critical difference in training detectors for use in BAS is the addition of translation augmentations. We translated each chip of 15 pixels in all ordinal and cardinal directions for a total of eight translations per chip. We continued our 5-degree rotation and flip augmentations for a total augmentation multiplier of 1296× and initialized our learning rate to 0.0001. Recall that the usage of data augmentation means that our single epoch of training was effectively 1296 epochs of training for variations of our training dataset.

### 2.4. Broad-Area Scanning (BAS)

In [35], it was shown that human broad-area search time can be variable and exceed numerous hours for large areas of interest (AOIs), even when searching for large-scale visual features in HR-EO-RSI. This is exacerbated as the geospatial footprint (extent) of objects continues to shrink. The value of machine learning technology in this domain is the reduction of human hours to accomplish cognitive tasks through human–machine teaming; in this case, this is accomplished by assisting human analysts in visual searches, i.e., machine-learning-assisted visual analytics. Therefore, it is critical to transition high-performing object detection models out of the experimental cross-validation settings and closed-world evaluations and into the application domain of aided imagery analysis tasks. Machine learning models, such as DCNNs, are ideal technologies to augment human analytical tasks, as the models do not fatigue and can simply scale with the data.

**Figure 2:**
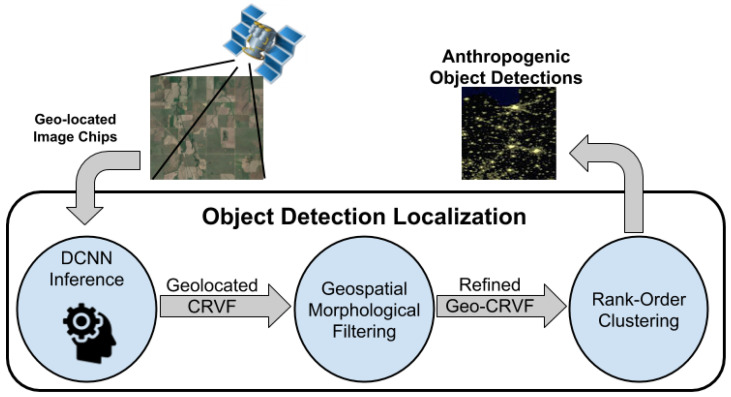
BAS with HR-EO-RSI for object detection and localization. Large imagery tiles are scanned in a highly overlapped method to stream image chips through DCNNs, thus resulting in a geolocated mesh of confidence vectors. The response surfaces are refined using morphological filtering, followed by mode-seeking clustering algorithms for localization.

An overall data flow for scanning broad areas of HR-EO-RSI using trained DCNNs is depicted in Figure 2. Large imagery tiles acquired from data providers are processed to generate a stream of geolocated imagery chips, which then flow through the DCNN to produce the CVRF. Following aggregation of the CVRF, candidate objects can be presented to users in an ordered fashion for rapid visual assessment. As a result if searching, for example, for anthropogenic objects such as baseball fields in a broad area, we can reduce the visual inspection to a few hundred image chips within the entire AOI. The remainder of this section details the algorithmic approaches to performing object localization in large-scale regions of Earth that have been scanned by machine learning models.

#### 2.4.1. Classification Vector Response Field

**Figure 3:**
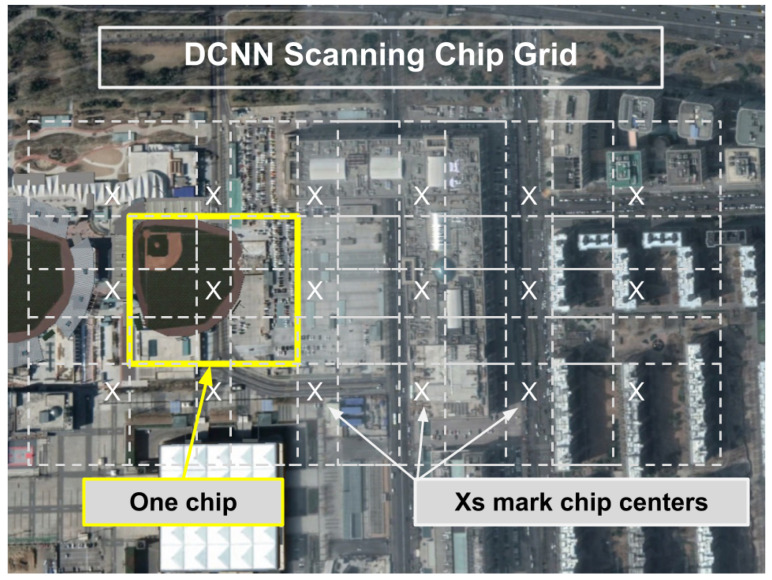
Imagery scanning with overlap for a single tile in a scanning AOI for DCNN inference. Chip centers are marked with X, and each chip is the size of the yellow box. Overlap is added to ensure all objects are detected regardless of the position in the tile.

The critical first step for BAS is the generation of the CVRF. It should be noted that the scale of satellite imagery and the area of analysis precludes the use of techniques such as region proposal networks or candidate object nomination non-maximal suppression, which are common in single image bounding box detectors. In this work, we leveraged an HR-EO-RSI provider to acquire two AOIs, the first of which was a quarter geocell and the second being a full geocell, with each covering 1 degree latitude by 1 degree longitude. All imagery were 0.5 m GSD multi-spectral imagery that were reduced to just the traditional color bands: red, green, and blue. The AOIs were scanned via a data collector that moved through each AOI to pull down large image tiles from the imagery provider’s web API. These image tiles were overlapped by 10% of their width, thereby ensuring sufficient AOI coverage by preventing objects from only being partially seen when captured on tile edges. Each tile was then processed with a stride of 90% of its width, which pulled image chips from the broad area that were 227 × 227 pixels in size (113.5 × 113.5 m). Figure 3 illustrates the chip scanning, which effectively forms a set of overlapping grids over the AOI. In the Beijing AOI, the larger of the two chosen for this study, our source imagery is composed of 33,201 PNGs at 1280 × 1280 pixels, meaning that each of our detectors must evaluate 54,396,518,400 unique pixels, which requires hours of processing even on high-performance NVIDIA V100S GPUs, due to memory limitations of GPU cards.

**Figure 4:**
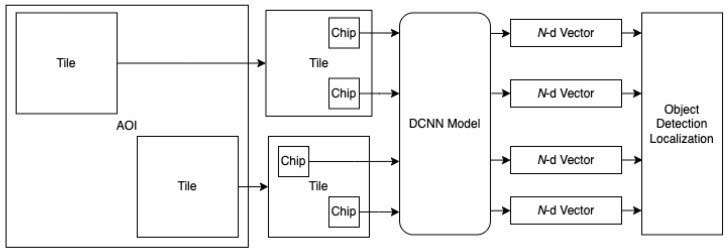
The AOI is scanned as a set of overlapping tiles, where each tile is processed as a set of overlapping chips. Each chip is passed through the DCNN to produce the geolocated classification vector (CVRF). The N-d vectors are then used as input for the object localization process. N is the number of classes upon which the model is trained.

Each image chip was pushed through the DCNN to generate a geolocated classification response vector. Figure 4 illustrates the relationship and data path from AOI tiles to chips to DCNN inference to classification vectors. The response vectors were automatically geolocated based on the chip center latitude and longitude (see also Figure 3). The CVRF generated from the AOIs in this research consisted of 601,344 geolocated classification vectors in the Springfield AOI and 11,983,927 vectors in the Beijing AOI.

The scanning forms an irregular field, i.e., mesh, of classification vectors over the AOI. A very important consideration is the nature of the classification output of the DCNN architectures. DCNNs typically utilize a softmax output layer that generates the final classification response. This layer type aids in the training of the DCNN. However, the effect is that the output vector, *C*, during inference is normalized such that

(1) $$1=\sum_{i=1}^{\left|C\right|}C_{i}.$$

Unfortunately, when the network has low activation because of a lack of presence of relevant visual content, as expected by the classifier, the vector response is lifted up to the normalized condition of Equation (1). Hence, the CVRF is expected to have densities of detections around true detections, as well as a massive number of artificially high responses that are false alarms (noise). Efficient processing of the CRVF to accommodate this real consequence of applying the DCNN in a real-world application setting is therefore a critical processing step.

#### 2.4.2. Multi-Object Localization

As just discussed, the CVRF cannot be taken as raw confidence in detections due to the softmax output layers on DCNN architectures. As such, we present an algorithm to perform multi-object localization across a broad area’s CVRF. The CVRF generated from the DCNN broad area scan can be accessed one classification layer at a time, thus effectively processing one response surface at a time. We refer to a single response classification layer, *R*, as a classification surface.

Based on DCNNs trained on the datasets used in this study, we yielded 21-layer, 38-layer, 45-layer, and 33-layer CVRFs for the UCM, PatternNet, RESISC-45, and MDSv2, respectively. The number of layers in a given model’s CVRF is equal to the number of classes in the dataset upon which the model was trained. Knowing this, we can, for each model, slice out a response field, *R*, for a single class, such as Baseball Field. This response field is then a surface over the AOI, therein having a topology shaped by a single DCNN output neuron. We can expect regions within the AOI that have probable baseball fields, in this example, to have a peak in *R*. *R* will, therefore, have some unknown number of peaks that we can discover and rank to achieve a broad-area search assisted by the DCNN. Recall, however, that the response surface may have many artificial spikes where the softmax layer has lifted up the confidence.

To discover the true detection peaks, we first apply an alpha cut using a confidence threshold. Recall that the output of the DCNN is a softmax layer; therefore, each output neuron produces a value in the range [0.0,1.0]. We used a threshold to drastically reduce our data space to only the points where the DCNN was most certain of the target class. In essence, we can produce a spatial field of densities where the dense regions will be the highest likelihood objects of interest. Afterward, we can seek the modes under the densities (localized peaks), and then we can label and rank the detection clusters.

Due to the nature of the softmax classification vectors from the DCNN, the density surface can have irregularities and holes when examined in the isolated context of a single-class surface, *R*, thus creating multiple small disparate densities (peaks) where, in fact, we expect a single larger density. Therefore, we seek to simultaneously smooth false alarms (outliers) and amplify the true detection densities for enhanced mode seeking. This is accomplished using a function-to-function morphology, with a distance decaying structuring function positioned over a detection response, i.e., the chip centers on the *R* that survive the alpha cut. We define our structuring function, s(p), as follows:

(2) $$s\left(p\right)=\left\{\begin{array}{cc} \exp^{\left(-d/D\right)}, & \mathrm{if}\;d<D;\\ 0, & \mathrm{otherwise} \end{array}\right.$$

where *p* is the chip position (center point), *D* is the maximum aperture of the structuring function (kernel), and *d* is the computed haversine distance of neighbor, *n*, as a function of *p*:

(3) d=haversine(n,p).

In this work, we constrained the aperture to D=150 m; however, this should be adjusted, in practice, based on various factors such as image resolution, target object size, and spatial distribution of image chip centers.

To generate the amplified spatial density surface, $R^{\prime }$, we apply s(p) to *R*, (Algorithm 1, step 3). Let N(p) be the spatial neighborhood of a point *p* having points *n*. Then, we define *p*’s value in the amplified spatial density field as the intersected volume defined by the spatial neighbors of of *p* and N(p), i.e.,

(4) $$\delta \left(s\left(p\right),N\left(p\right)\right)=\sum_{n\in N(p)}\max\left\{R\left(p\right),R\left(n\right)\right\}*s\left(p\right).$$

**Algorithm 1:** Spatial Clustering of Classification Vector Response Field (*R*)

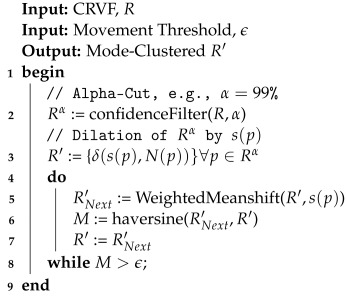



**Figure 5:**
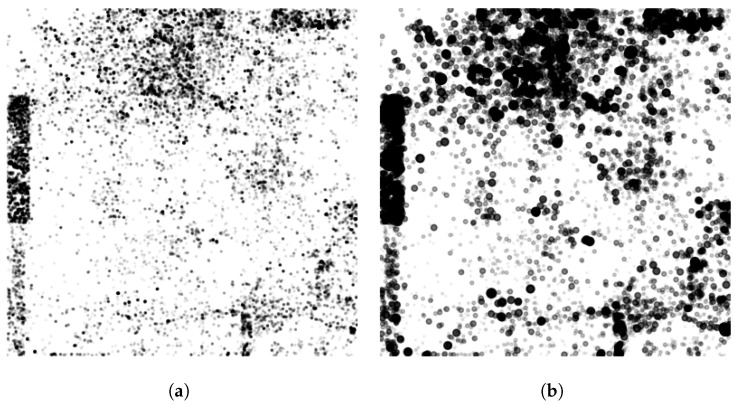
AOI response surface for a UCM-trained EfficientNet model. (**a**) Each dot is a location in the AOI in which the model’s confidence of storage tank is ≥0.99. (**b**) The amplified spatial density surface following function-to-function morphology with a distance decaying structuring function.

Mode-seeking clustering algorithms are designed to discover the number of clusters (or peaks) without relying on the specification of expected clusters ahead of time. This is an ideal approach for refining broad-area scans of HR-EO-RSI, as we do not know *a priori* the number of objects we expect to find. In the case of $R^{\prime }$, the clusters are the modes within the field that are the local maxima. These maxima are peaks found as the center of mass of spatially connected densities in $R^{\prime }$. A straightforward approach to discovering these modes is the mean shift algorithm, which defines a spatial aperture of the nearest neighbors to be evaluated at each iteration for each point. Each point is then moved to the center of mass of its spatially local neighbor set. Algorithm 1, steps 4–8, provides the algorithm to mode cluster $R^{\prime }$. The algorithm defines the processing of a classification response *R* from the CVRF into the amplified spatial density surface, $R^{\prime }$, which then leads to the evolution of the $R^{\prime }$ into clusters representing object detections. A sample AOI response surface and amplified spatial density surface can be seen in Figure 5.

In this work, we utilized a variant of the mean shift algorithm that operated in both the spatial proximity and amplified spatial field domain, which was labeled as the *weighted meanshift* in Algorithm 1, step 5. The spatial decay function, Equation (2), was used to weight the computation of the local field mean density. We defined the *weighted meanshift* as the standard mean shift algorithm, thereby computing the weighted distance between $p\in R^{\prime }$ and n∈N(p) as d(p,n)=haversine(p,n)∗s(p). Points were continually evaluated and shifted until the total Earth surface movement of all the points was less than one meter. As above, we used 150 m as the aperture radius (i.e., neighborhood) around each point during weighted mean shift evaluation.

**Figure 6:**
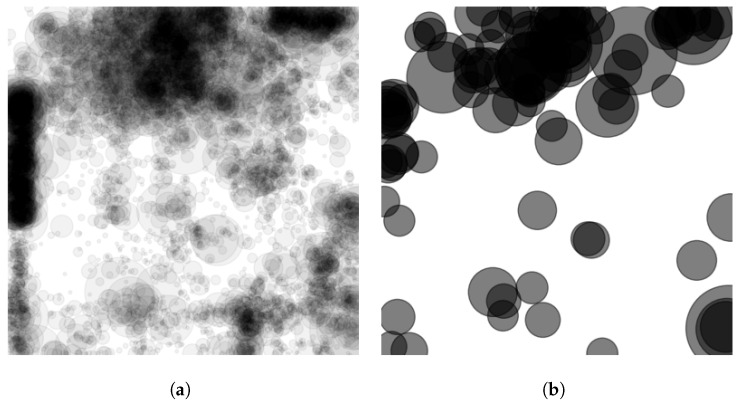
Clustering results from the UCM-trained EfficientNet model. (**a**) Clustering results, where the darker areas indicate higher-ranked final detections. (**b**) top 100 cluster locations with larger clusters ranked higher. Rank is indicated by the size of the circle.

Once the points have converged under the modes, they are then mapped onto their respective clusters, which are labeled and ranked. Each cluster’s score is computed as the volume under its amplified spatial density. The cluster score can then be used to rank (highest to lowest score) all clusters in an AOI to determine the order for subsequent human analysis. Algorithm 2 provides the algorithm with the means to extract the rank-ordered clusters from the mean-shifted data. In this study, single-detection clusters (i.e., spatial outliers) were filtered out from the results. Figure 6 shows the result of the clustering along the application of a top 100 cluster limit.
**Algorithm 2:** Object Detection Ranking ($R^{\prime }$)
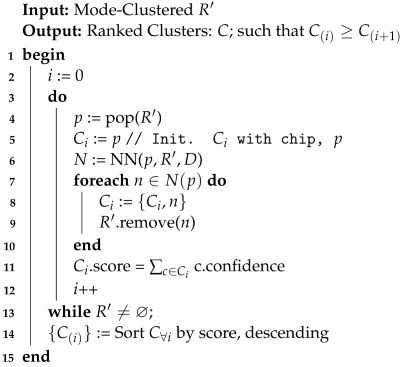


## 3. Results and Discussion

Most research literature evaluates machine learning models solely in the context of well-constructed, discrete datasets, i.e., the closed-world model. In this setting, well-understood cross-validation methodologies are typically used. For instance, many dataset authors evaluate the baseline performance of numerous machine learning approaches using a five-fold or ten-fold cross-validation on their newly published datasets (e.g., [3,4,5,6]). Therein, a single fold is withheld from the model training phase, which is then used as blind validation data. This is repeated for each fold until every data fold is withheld from training and then used for validation.

However, an open question is how well models that are high-performing on discrete datasets will adapt to the complex realities of broad area HR-EO-RSI. As it so happens, rarely are tools applied to satellite imagery in the context of small image chips of a few thousand pixels. Real-world remote sensing data are truly big data, and they often exceed hundreds of millions of pixels. However, one of the greatest challenges regarding the evaluation of machine learning models taken out of the clean laboratory setting and into the real world is the difficulty of measuring performance. DCNNs applied to the remote sensing domain are no different. In the real world, the ever-changing anthropogenic landscape of Earth ensures that any active scanning of the large swaths of HR-EO-RSI is sure to discover new objects that cannot be efficiently cataloged by humans into a ground truth database.

In this section, we establish the baseline model performance for three leading deep neural network architectures across a collection of benchmark datasets. Then, we propose a metric inspired by the field of *information retrieval* (see [36,37]) to allow for the comparison of the models in an applied remote sensing data processing task. We then examine how the various network architectures performed in BASs relative to each other. This provides a foundation for discussing the cross-validation versus scanning performance, thus offering insights into the generalizability of the respective architectures, as well as the suitability of the datasets for training real-world models.

### 3.1. Cross-Validation Performance

The cross-validation performance of the three DCNNs (Section 2.2) trained with the four benchmark datasets (Section 2.1) provides a baseline of the expectation for performance. Unfortunately, these discrete, well-partitioned datasets are not representative of the real-world operating environment. Nevertheless, we must understand the *clean room* operating characteristics to effectively evaluate the transition into the real world. Five-fold cross-validation was run using the training methodology detailed in Section 2.3.1. The results of these experiments are shown in Table 3.

The results of cross-validation show that all of our networks were able to classify the four chosen datasets with high levels of recall and precision. The lowest score seen in cross-validation was the 95.72% F1 Score obtained by ProxylessNAS in classifying RESISC-45, which is a very respectable score, given the heterogeneity of the dataset. Looking across datasets, we see that UCMerced and PatternNet were easier to classify than MDSv2, which was then more easily classified than RESISC-45. This trend remained consistent across all of the three evaluated networks.

Comparing results across networks, Xception seemed to slightly outperform ProxylessNAS for all four datasets, but only by less than half a percent. Xception was then, in turn, outperformed by EfficientNet on all four datasets by varying margins. EfficientNet outperformed Xception by less than half a percent on the less challenging datasets of UC Merced and PatternNet, but then that gap in the F1 Score grew to over half a percent for the harder datasets of MDSv2 and RESISC-45. Margins larger than half a percent are a more significant margin when scores are higher, as an increase from 95.82% to 96.47% on the RESISC-45 F1 Score is a reduction in error of over 15%.

### 3.2. Scanning Metrics

As discussed, it is nearly impossible to judge how well high-performing models, measured in the context of discrete datasets, will perform for applied remote sensing tasks. Given that many of the contemporary deep neural network models have performed in excess of 95% on numerous datasets, it is necessary to develop evaluative procedures to compare the applied performance of these models on full remote sensing data with real-world contextual and compositional complexities. For instance, if examining the top twenty candidate object detections from an applied broad-area search with two separate DCNNs, how do we evaluate the relative worth of each model if both DCNNs had one false detection in the top 20? In traditional machine learning metrics, this is the *true positive* (TP) divided by the number of TP plus *false positives* (FP). In other words, the ratio of the result set that was relevant, which in both cases of this example is 95%. However, what if the first DCNN had the FP at rank 1, and the second DCNN had the FP at rank 20? We should have a metric that indicates that the first DCNN (rank 1) is performing worse than the second DCNN (rank 20), since its false positive is the highest-ranked detection.

For this reason, we propose a metric that allows for the relative measure of success between two models or scanning algorithms applied to large areas for an object discovery task when acquiring ground truth is intractable. Let $T=\{O_{1},O_{2},\ldots O_{\infty }\}$ be a set of an unknown quantity of relevant objects that exists within the broad area. Let $L=(O_{\left(1\right)},O_{\left(2\right)},\ldots O_{\left(N\right)})$ be a ranked object candidate list generated by a scanning method (i.e., model and algorithm for localization). Then, a measure of the scanning precision for *L* is defined as follows:

(5) $$P_{scan}\left(L\right)=\sum_{i=1}^{m}\frac{i}{r(L,i)},$$

where $m=\min(|L|,n_{r})$ and r(L,i) represent the rank of relevant (true) detection *i* in the set *L*. $n_{r}$ is the number of true detections discovered *a posteriori* in *L*. Of note, the *a posteriori* is captured by *m* in Equation (5), which can be set based on ground truth or based on the union set of detected objects from multiple model scans.

**Figure 7:**
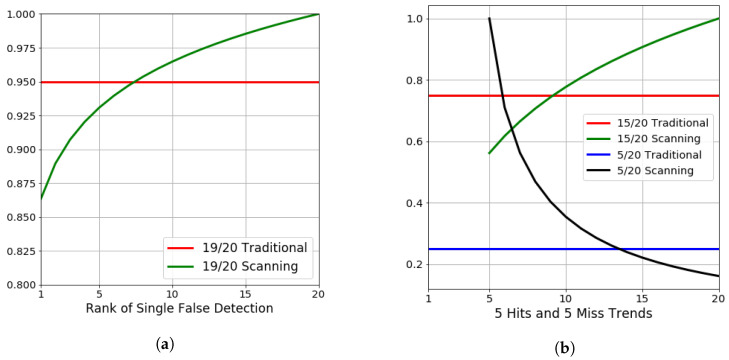
Scanning precision metric compared to the traditional precision metric. Note how the scanning precision more heavily penalizes missed detections (false positives) at higher ranks and penalizes missed detections at lower ranks less. (**a**) Geometric representation of a single false detection as the missed detection occurs at different ranks. (**b**) Geometric representation of 5 correct and 5 missed detections as the misses move between higher and lower ranks.

The characteristic of this metric is based on the idea that algorithms and models that are more discriminatory and allow true object detections to rank higher in a result set are better. Figure 7a demonstrates the metric of Equation (5) as the non-relevant result moves lower and lower in the rankings. For comparison, the horizontal line represents the traditional 95% accuracy that would be reported for all scenarios.

Figure 7b also includes the complement of only relevant objects in the set, with the trend shown that the five relevant objects slide lower and lower in ranking. It also provides the 25% traditional precision for reference. It should be noted that the metric penalizes the result with the higher-ranked non-relevant results when comparing two ranked lists of the same size. Finally, note that when there are only five objects that are known *a posteriori*, as the rank of those items slides down the list, the scanning metric exponentially decays.

While limiting false positives at high ranks following post-processing may be preferred for several applications, there are also situations in object detection where limiting false negatives (FN) is far more important, and, for those applications, the scanning precision metric is not the ideal metric to evaluate the model performance. In most situations, it would be impossible to measure the number of false negatives during BASs, because the number of locations of the ground truth objects of interest is unknown. However, because we have obtained the ground truths for both of our scanning AOIs for three separate object classes of interest, we were able to report a scanning recall (SR) metric. The SR metric is defined as TP/(TP+FN), and it is identical to recall in traditional classification tasks. By presenting both an SP and SR, we can better understand the FP versus FN tradeoffs of our models, as well as how the SP relates to traditional metrics, such as the F1.

### 3.3. Springfield AOI Localization Performance

**Figure 8:**
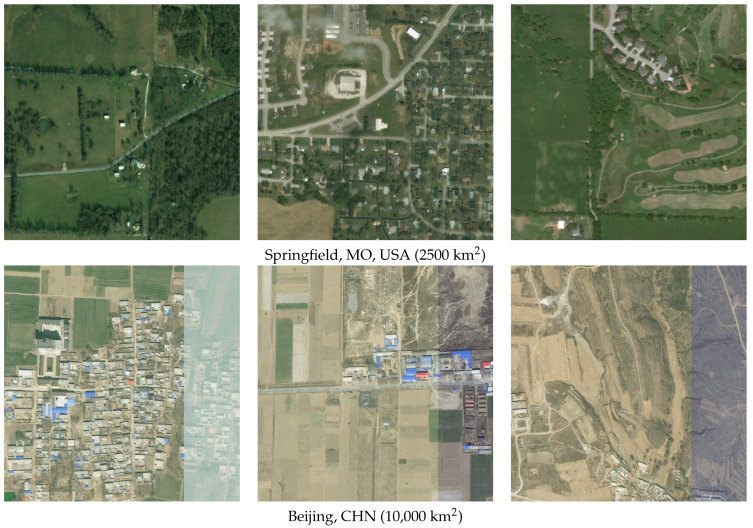
Sample imagery tiles from each of the scanning AOIs. Imagery are RGB and downloaded as overlapping tiles at 0.5 m GSD.

To measure object detection localization performance, full benchmark datasets were used to train new DCNN models (see Section 2.3.2). In other words, all available data that had appropriate labels were used to train the networks used for BAS, as opposed to the 80% used for each fold in the cross-validation experiments. We chose to utilize the EfficientNet-B4 network for the BAS, due to its superior performance shown in cross-validation and its excellent trade-off between model performance and GPU inference time. We then performed scanning on two geographically distinct AOIs: a one-quarter geocell AOI surrounding Springfield, Missouri, USA and a full geocell AOI surrounding Beijing, CHN. Sample imagery from each of these AOIs can be seen in Figure 8.

We begin our BAS performance discussion with model performance in the Springfield AOI. Each broad area scan in the Springfield AOI involves processing 9396 tiles, which are then chipped into 601,344 chips at 227 × 227 with a stride of 57 pixels. As highlighted in Algorithm 1, the CVRF is sliced to extract a single classification response surface, containing nearly 12 million points, for each of the three investigated object classes.

Table 4 provides the per-class number of object detections that survived the 95% confidence alpha cut, as well as the number of cluster centers following post-processing alongside the SR and SP. For reference, we also provide the mean F1 score achieved by the EfficientNet models for the same set of classes in Table 5. Of note, the number of object detections was significantly large to the point of clearly over-classifying patches of Earth during the scanning for some of the models, which depended on the training dataset. Both our SP and SR require a list, *R*, that describes which detections in the ranked list of candidates are true detections, i.e., they are valid. This list was obtained through Algorithm 3 with a buffer of 200 m, and it was then fed into the formulae for SP (Equation (5)) and SR.
**Algorithm 3:** Scoring of Cluster Centers
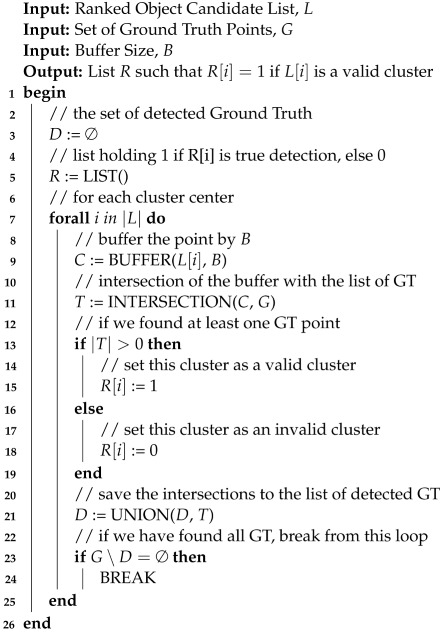


We now review, first on a class-by-class basis and then in summary, the results of the Springfield BAS experiments:

#### 3.3.1. Airplane

The first takeaway from the airplane results (Table 4) is the high range of pre-processing hits, with as few as 10 and as high as 1222, reported by the models trained on the PatternNet and MDSv2 datasets, respectively. This range in hits was replicated in the number of detections, with only a single detection coming from the model trained on the PatternNet dataset and 239 detections being derived from the model trained on MDSv2. We can observe that the model with the fewest reasonable number of detections achieved the highest SP, thus indicating that more discriminative models detect fewer false positives. However, the airplane results showed overall poor SP performance, with the highest SP reaching only 29%, which was achieved by the model trained with the UC Merced dataset.

Upon reviewing the SR results for the airplane class, we see that the most balanced model was trained on the RESISC-45 dataset, considering that its gap between the SP and SR was only 2.5%, while that gap was significantly larger for the models with the highest performance in each statistic. The best-performing SP model, trained on the UC Merced dataset, had an SP of 29%, but only a 9.4% SR, while the best-performing SR model, trained on MDSv2, had a 56.3% SR, but only a 6.2% SP. This trend indicates that the choice of the training dataset for aircraft detection in the Springfield AOI is dependent upon the sensitivity to FPs and FNs.

#### 3.3.2. Overpass

The overpass results, seen in Table 4, showed overall better model performance than the airplane class, thus indicating that these overpasses are perhaps not as heterogeneous in the Springfield AOI. We again see that there was a large disparity in the number of hits and detections between the models trained on different datasets, with 14 detections yielded by the model trained on UC Merced and 789 yielded by the model trained on MDSv2. The models were overall more balanced in detecting overpasses between SP and SR, as two of the four models were within a 3.5% difference between the SP and SR. The results for overpasses are more competitive than for airplanes, with the SR scores being much closer between the models trained on disparate datasets. Unfortunately, none of the models were able to achieve SP or SR scores that were over 50% for the overpass class, with the best SP achieved by the model trained on UC Merced at 46.1% and the best SR achieved by the model trained on MDSv2 at 34.2%.

#### 3.3.3. Storage Tank

The storage tank results, shown in Table 4, were similar to previous classes’ performance results, with large disparities in the number of hits and detections that ranged from 48 hits (PatternNet) to 5892 hits (MDSv2), which is an egregiously high number of hits in a 2500 square kilometer area for a single class. We again see large gaps between the SR and SP scores for all but one model (trained on UC Merced), which again suggests that models trained on these datasets must be specialized either for the minimization of FNs or FPs according to the nature of the dataset. For both the SR and SP, the best performance was around 60%, although those scores were achieved by different models, with the best SP reported by the model trained on PatternNet and the best SR reported by the model trained on MDSv2.

#### 3.3.4. Summary of Springfield BAS Performance

Looking across the results of all the classes in the Springfield AOI, we see several interesting trends. First, we see that the best overall score reported by any model on any class was 59.4%, which was the SR achieved by the MDSv2-trained model on the Storage Tank class. No model was able to achieve a 60% SP or SR for the Springfield AOI, which would suggest that these models are not well-tuned for detection in the Springfield AOI, and it is unclear as to whether that is due to the training datasets or the training methodology. Secondly, while the best SP across the three classes changed between the models trained on the UC Merced and PatternNet datasets, the best SR for all three classes was achieved by the model trained on MDSv2. Recall that the MDSv2 is a collection of overlapping classes from six other datasets, including UC Merced, PatternNet, and RESISC-45. This result indicates that the increased intra-class variation achieved by combining these six datasets enables models to detect more objects; however, this may come at the cost of more FPs, as per the low SP scores reported by the model trained on MDSv2. The last major takeaway from the Springfield AOI experiments is the surprisingly competitive SP scores reported by the model trained on the UC Merced dataset. The UCMerced dataset is the oldest and smallest dataset in this study at only 2100 samples, which are only 2.41% the number of samples in the largest dataset, MDSv2, but the EfficientNet model trained on UC Merced was able to achieve the best SP on the Airplane and Overpass classes. This observation may be a case of over-fitting or over-specialization of the network on the Airplane and Overpass classes; however, if these objects are important to a human analyst conducting a BAS, then this is the ideal training configuration for this model.

### 3.4. Beijing AOI Localization Performance

We now move on to the Beijing AOI BAS Experiments. These experiments were conducted with the same trained models from the Springfield AOI, and the BAS, along with post-processing, were identical aside from a raising of the required confidence threshold during post-processing from 95% to 99% to account for the much larger size of the Beijing AOI. The scoring was conducted identically to the Springfield AOI: raw detections were run through weighted mean shift post-processing, and those results were then programmatically reviewed with previously attained ground truths using Algorithm 3, which was then used to calculate our SP and SR. The resulting scores and counts of the hits and post-processed detections can be found in Table 6.

We now review, on a class-by-class basis, the results of the Beijing BAS experiments using the EfficientNet-B4 model.

#### 3.4.1. Airplane

The ability of the detectors to accurately detect airplanes varied between detectors; however, even the best SP of 33.2%, reported by the UC-Merced-trained detector, indicates that our models are unable to detect airplanes without a significant number of FPs. This idea reinforces the Springfield AOI results, which also saw low SP scores for the Airplane class. Unlike the Springfield results, however, our detectors were able to achieve a respectably higher score for the SR in the detection of airplanes. While the MDSv2-trained model was able to detect airplanes with the same level of recall as the Springfield AOI, the RESISC-45-trained model outperformed the rest of the detectors and was able to detect 86.9% of the 107 ground truth airplanes in the Beijing AOI. Although we see an excellent SR by our MDSv2- and RESISC-45-trained models, this did come at the cost of a much lower SP of 2.7% and 12.5%, respectively. This means that, for applications that are more sensitive to FNs, these models may be a good choice, but they would be inadequate for applications that are more sensitive to FPs.

#### 3.4.2. Overpass

Upon reviewing the overpass scores, displayed in Table 6, we see that three of our four detectors were able to detect overpasses with at least a 60% SR, with two of our models achieving SR scores above 80%. The best overall models, trained on the MDSv2 and RESISC-45 datasets, not only managed to achieve a 60% or higher SR, but they did so with a 57% or higher SP, which was the best combined performance in this study, regardless of target or AOI. Again, we see that the model trained on PatternNet lagged in performance compared to the other three models, thus indicating that this dataset is not optimally designed for the detection of overpasses in BAS applications.

#### 3.4.3. Storage Tank

While our models had inconsistent performance results in detecting other objects with high levels of SR, all four of our models were able to detect storage tanks in the Beijing AOI with at least 61% SR when trained on the PatternNet, and as high as 96–97% when trained on the UC Merced and MDSv2 datasets, respectively, as shown in Table 6. The deterministic and homogeneous visual appearance of storage tanks may be contributing to the models’ high SR scores in the Beijing AOI. Unfortunately, the excellent SR values reported by our models came at the cost of precision, as three of our four detectors reported SP scores below 30%, thus showing a high number of FPs among the TPs detected in our AOI.

## 4. Conclusions

In this work, we presented the methodologies for performing anthropogenic object detection with deep convolutional neural networks (DCNNs) in aerial imagery. We selected four open source HR-EO-RSI datasets and showed the DCNNs’ ability to excel in the classification of those datasets.

We then performed BAS experiments across two geographically distinct AOIs using models trained on the four open source high-resolution datasets and scored our models with previously acquired ground truth data. We presented a metric adapted from the information retrieval domain for scoring our detectors in BAS experiments, both with and without ground truth information. We observed an inherent tradeoff between the FPs and FNs for nearly every combination of AOI, target class, and training dataset, which suggests that each application of the DCNNs requires the specialized tuning of training parameters and techniques according to the requirements of that application. Most importantly, the outcomes of our BAS experiments showed the inability of the cross-validation experiments to accurately assess a model’s ability to perform with the non-closed set, real-world data, such as the BAS task. The F1 scores for the trained models on the selected classes in cross-validation were in excess of 96%, and yet these same models struggled to detect those same classes above a 5% SR in some instances. The contradiction between the BAS scores and cross-validation scores indicates that the selection of a model for BAS applications cannot be performed based solely on the cross-validation scores of the target classes.

Our future work for this research includes expanding our work into larger AOIs and searching for more target classes. Additionally, the release of more sophisticated neural networks offers an opportunity to better detect the classes of interest in BAS applications.

## Figures and Tables

**Table 1:** Number of Images in Classes of Interest for UCMerced (**UCM**), PatternNet (**PN**), RESISC-45 (**R-45**) and Improved MetaDataset (**MDSv2**).

Class	UCM	PN	R-45	MDSv2
Airplane	100	800	700	2545
Baseball Diamond	100	800	700	1600
Overpass	100	800	700	1658
Storage Tanks	100	800	700	10,042
Tennis Court	100	800	700	1600

**Table 2:** Number of collected ground truth objects (**Ct.**) in selected classes in the Beijing and Springfield AOIs along with the human time (H:M:S) required to scan each AOI for each target class (**Time**).

	Springfield AOI		Beijing AOI	
Class	Ct.	Time	Ct.	Time
Airplane	32	32:17:17	107	12:29:11
Overpass	79	36:01:04	773	16:20:25
Storage Tank	244	3:37:04	591	12:59:31
Total	355	71:55:25	1471	41:49:07

**Table 3:** Cross-validation performance of selected network architectures over the investigated datasets. Each network architecture underwent five-fold cross-validation with all 4 selected datasets. The metrics shown are average values across all five folds for each dataset.

Network	Dataset	Recall	Precision	F1 Score
EfficientNet	UC Merced	98.67	98.72	98.66
	PatternNet	99.70	99.70	99.70
	RESISC-45	96.47	96.52	96.47
	MDSv2	97.91	98.05	97.97
ProxylessNAS	UC Merced	98.24	98.34	98.24
	PatternNet	99.48	99.50	99.48
	RESISC-45	95.73	95.79	95.72
	MDSv2 [26]	97.24	97.38	97.29
Xception	UC Merced	98.57	98.63	98.57
	PatternNet	99.58	99.58	99.58
	RESISC-45	95.83	95.90	95.82
	MDSv2	97.32	97.34	97.31

**Table 4:** Scanning results of anthropogenic object localization in Springfield AOI, including post alpha cut counts (**Hits**), number of cluster centers (**Clusters**), the recall of each class in the AOI (**SR**), and the scanning precision for the clusters (**SP**).

Dataset	Class	Hits	Clusters	SR	SP
UC Merced	Airplane	266	51	9.4	29.0
	Overpass	52	14	6.3	46.1
	Storage Tank	1228	229	29.9	30.6
PatternNet	Airplane	10	1	0.0	0.0
	Overpass	872	118	31.6	28.4
	Storage Tank	48	9	2.0	58.9
RESISC-45	Airplane	375	63	15.6	18.1
	Overpass	273	43	25.3	45.3
	Storage Tank	153	26	7.8	40.6
MDSv2	Airplane	1222	239	56.3	6.2
	Overpass	789	115	34.2	34.9
	Storage Tank	5892	1001	59.4	29.7

**Table 5:** Per-class F1 Score for selected classes trained with EfficientNet during cross-validation. These same models were used for BAS. Note the excellent cross-validation performance of the models on all three investigated target classes.

Class	UCM	PN	R-45	MDSv2
Airplane	99.48	100	98.71	99.15
Overpass	99.02	99.87	96.96	98.33
Storage Tank	97.48	99.94	98.36	97.48

**Table 6:** Scanning results of anthropogenic object localization in Beijing AOI, including post alpha cut counts (**Hits**), number of cluster centers (**Clusters**), the scanning recall (**SR**) of each class in the AOI, and the scanning precision for the clusters (**SP**).

Dataset	Class	Hits	Clusters	SR	SP
UC Merced	Airplane	692	101	16.8	33.2
	Overpass	70,635	8305	94.8	28.8
	Storage Tank	53,493	6461	96.3	15.6
PatternNet	Airplane	116	19	5.6	12.2
	Overpass	5884	816	38.6	32.7
	Storage Tank	3619	483	61.4	42.9
RESISC-45	Airplane	5222	902	86.9	12.5
	Overpass	5958	637	64.9	77.8
	Storage Tank	5440	682	84.9	29.1
MDSv2	Airplane	16,430	1219	58.9	2.7
	Overpass	13,626	1729	83.7	57.9
	Storage Tank	142,996	17,131	97.0	12.0

## Data Availability

Publicly available datasets were analyzed in this study. This data can be found here: UCMerced: http://weegee.vision.ucmerced.edu/datasets/landuse.html, accessed on 2 May 2023; PatternNet: https://sites.google.com/view/zhouwx/dataset, accessed on 2 May 2023; RESISC-45: http://www.escience.cn/people/JunweiHan/NWPU-RESISC45.html, accessed on 2 May 2023; MetaDataset: https://github.com/scottgs/metadataset_objects, accessed on 2 May 2023.
